# Is access to intensive care equitable?

**DOI:** 10.1186/s13054-018-2203-x

**Published:** 2018-11-03

**Authors:** Claudio M Martin

**Affiliations:** Division of Critical Care Medicine, Department of Medicine, Schulich School of Medicine & Dentistry, Victoria Hospital, London Health Sciences Centre, Western University, 800 Commissioners Rd E, London, ON N6A 4S2 Canada

Patients with life-threatening illness often require admission to intensive care units (ICUs) to receive life sustaining interventions. This is an expensive health care resource with specialized physical space, equipment and healthcare worker expertise. Due to constraints imposed by a limited resource, various guidelines for admission to the ICU have been proposed [1], including triage decisions if demand exceeds supply [2]. Institutions likely have their own more specific admission and discharge criteria for individual ICUs. These guidelines are subject to interpretation and, especially when combined with individual treatment decisions, introduce the possibility of subjectivity and bias. Whether such inequities in access to ICU care exist is not clearly known, or easy to study.

A prospective, multicentre, cohort study from Europe found age but not gender was an independent predictor for refusal of admission to ICU [3]. A population study using a large administrative dataset in Ontario, Canada reported that female sex was associated with lower likelihood of ICU admission and fewer ICU interventions [4]. Another population study using administrative data of hospitalized patients reported men were more likely to receive ICU admission and invasive mechanical ventilation but women who received ventilation were more likely than men to have non-invasive ventilation [5]. Females were less likely to receive early, goal-directed treatment (defined as measurement of ScVO2 through central line) in a single centre cohort study [6]. Although this latter study was conducted in an emergency department, it may be considered an ICU-level intervention. Female sex was also associated with the decision to forego life-sustaining therapies once admitted to the ICU [7]. Race, culture and ethnicity are other potential biases that might influence decisions to provide ICU care. In a secondary analysis of a prospective cohort study, black adults were less likely than white adults to be admitted to a coronary care unit (CCU) and had fewer CCU days when they were admitted [8].

To ensure that healthcare access is equitable for all members of our populations, it is important to have valid methods to measure this. This is not a simple task, since the admission process is complex. ICU admissions usually come from the ward, operating room or emergency department and require a referral from the appropriate physician to the intensivist, who must then assess if the patient meets the ICU admission criteria. Critical care rapid response or medical emergency teams may influence this process in hospitals where they exist. In a study by Garland and coworkers [9], the authors assert that multiple factors can affect the decision for the patient to arrive at the hospital in the first place, which is an idea based on Andersen's Behavior Model [10].

Population-based studies are likely to become more prevalent and can provide important insights. For example, it was recently reported that recent immigrants received more aggressive end of life care than long-standing immigrant residents [11]. Associations made from observational studies will always be subject to unrecognized and unmeasured residual confounding. When rates are examined and compared, it is vital to select the appropriate denominator. Garland and coworkers [9] propose that this denominator should be the number of patients eligible for ICU care in the community for two main reasons. First, the general population is not the appropriate denominator since the predisposition to critical illness is not uniform. Second, a hospital-based denominator is not appropriate since potential biases leading to inequitable access may be expressed at any point in the illness trajectory, including the decision to consider hospital admission. Since a prevalence measure of critical illness or predisposition to ICU in the general population is not available, they explored the use of population-based palliative care deaths as a surrogate for critical illness and potential ICU admissions. With this approach, the apparent bias for ICU admission due to sex disappeared while noting lower ICU admission for people in lower income strata.

Population health studies are largely based on administrative data and therefore dependent on the accuracy of the coding and algorithms used for analysis. Themes such as palliative care provided in the community or in hospitals are based on coding methods and errors that will vary between institutions, regions and countries. The truth of course lies somewhere within this bubble of uncertainty. Studies of access to ICU also must consider that critical care is a system within the larger hospital and health care system, as modelled by the Garland et al. study [9]. Despite methodological limitations, we must acknowledge that many social, environmental and population factors affect access to critical care. Despite conflicting evidence, female sex appears to be one of these factors. Addressing these inequities to access will require interventions across the entire healthcare system. Progress can only be measured using the tools available to us, which hopefully will also continue to improve.
